# Assessment of childbirth preparation classes: a parallel convergent mixed study

**DOI:** 10.1186/s12978-019-0826-2

**Published:** 2019-11-07

**Authors:** Robab Hassanzadeh, Fatemeh Abbas-Alizadeh, Shahla Meedya, Sakineh Mohammad-Alizadeh-Charandabi, Mojgan Mirghafourvand

**Affiliations:** 1 Students’ Research Committee, Tabriz University of Medical sciences, Tabriz, Iran; 2 Reproductive Health Research Center, Tabriz University of Medical Sciences, Tabriz, Iran; 3 South Asia Infant Feeding Research Network (SAIFRN), School of Nursing, Faculty of Science, Medicine and Health, University of Wollongong, Wollongong, Australia; 4 Social determinants of Health Research Center, Midwifery Department, Tabriz University of Medical sciences, Tabriz, Iran

**Keywords:** Childbirth preparation classes, Antenatal education, Birth experience, Mixed method

## Abstract

**Background:**

Women’s fear from childbirth has been associated with increased medical interventions and traumatized birth experience. Although antenatal education is a crucial factor to empower and prepare women for their birth journey, it is not clear how Iranian childbirth classes can influence women’s fear and prepare them positively towards childbirth. This research is designed to evaluate childbirth preparation classes and their impact on women’s perception on their childbirth experiences.

**Methods/design:**

This mixed method study with the parallel convergent design has two phases. The first phase will be a quantitative cohort study with 204 primiparous pregnant women at the gestational age of 35–37 weeks. The participants will be divided into three groups based on the number of their attendance into the childbirth preparation classes: a) regular participation (4 to 8 sessions), b) irregular participation (1 to 3 sessions), and c) no-participation. Participant will be followed-up to 1 month after birth. Antenatal data will be collected by using a demographic survey questionnaire, the Wijma Delivery Expectancy/Experience Questionnaire (W-DEQ, version A), the Van den Bergh Pregnancy-Related Anxiety Questionnaire, the Satisfaction with Childbirth Preparation Classes Questionnaire, the Edinburgh Postpartum Depression Scale (EPDS) and Knowledge regarding pregnancy and childbirth Questionnaire. Postnatal data will be collected by using an Obstetric and Labor Characteristics Questionnaire, EPDS, and Childbirth experience questionnaire (CEQ). The quantitative data will be analyzed using one-way ANOVA and the multivariate linear regression. The second phase of the study will be a qualitative study that will explore the women’s perceptions on the impact of participation in childbirth preparation classes on their childbirth experience. The sampling in this phase will be purposeful and the participants will be studied individually by using in-depth, semi-structured interviews. The qualitative data will be analyzed through content analysis with conventional approach.

**Discussion:**

Assessing the impact of childbirth preparation classes on women’s childbirth experience in Iran will lead to developing recommendations about the content and quality of the childbirth classes that can improve women’s’ preparation towards positive childbirth.

## Plain English summary

Fear of unknown in pregnant women increase complication of the pregnancy, labor and consequently the medical intervention. Pregnant mothers often need prenatal education to decide on their childbirth options such as positions during labor, pain relief methods, infant care and breastfeeding. Participation in childbirth classes can increase women’s level of knowledge regarding childbirth and potential effect on anxiety and fear about childbirth experience. However, in Iran, a very limited number of quantitative studies have been performed to identify the relationship between participation in childbirth classes and the fear of delivery. The current study is designed to evaluate childbirth preparation classes on women’s fear and their preparation towards a positive childbirth experience. This study is a mixed-method with the parallel convergent design. There are two phases. Phase one is a quantitative cohort study in which the status of women’s childbirth fear, prenatal and postpartum depression, anxiety, knowledge regarding pregnancy and child birth, and satisfaction with childbirth preparation will be measured. The outcome measures will be compared among the women with regular participation, irregular participation, and no participation in childbirth preparation classes antenatally and postnatally. The qualitative part of the research will explore the women’s perceptions of the impact of their participation in childbirth classes on their childbirth experience and the perception of fear.

## Background

Childbearing is an important event of a women’s life [1]. Childbirth is a multidimensional process with physical, psychological, emotional, social, and cultural dimensions. At the same time, it can be accompanied by fear and concern [2]. Childbirth preparation education plays an important role in the physical and psychosocial preparedness of the mother [1, 3]. Pregnant women often attend prenatal education to decide on their childbirth choices, learn about different situations during labor, pain relief methods, infant care, postnatal care, breastfeeding, and parenting [4, 5].

Although, childbirth education has a long history in midwifery, Childbirth without fear came to the attention of Dick-Read, a British obstetrician who advocated natural childbirth. Dick-Read believed that excessive pain in labor resulted from muscular tension arising from fear of the birth process and giving information about the labor process and increasing women’s awareness, as well as providing practical relaxation training, could reduce fear of childbirth and, consequently, decrease labor pain. In the 1940s, the “Read” method, which emphasized pain relief by giving information on childbirth, formed the basis of childbirth education [6, 7].

In Iran, the routine prenatal care was limited to regular examinations, routine tests, and ultrasound. However, this routine care was not adequate to enhance women’s knowledge on how a baby is born and how mothers can make childbirth more comfortable for themselves. Lack of awareness and control led to increased anxiety and complications and, hence, increased medical interventions such as demand for elective cesarean sections [8–10].

Pregnant women experience anxiety about labor, labor pain, and the health of their unborn babies, but sometimes anxiety can take the form of an illness and affect their mental health [11–15]. Pregnant mothers’ fear and anxiety can lead to problems such as early delivery and low birth weight [16]. Increasing women’s awareness through education and counseling during pregnancy can reduce maternal fear and anxiety, prepare them for delivery and promote their health [6, 17]. In addition, the information received and the extent of involvement in the decision-making process can affect the woman’s childbirth experience. The all-encompassing experience that women gain from the delivery process is considered one of the most important outcomes of childbirth that will remain with them throughout their lives [18]. Studying women’s experiences of childbirth helps caregivers to better understand the needs and expectations of pregnant mothers and perform effective interventions based on the needs of these women to increase their satisfaction [19, 20]. On the other hand, negative birth experience can influence women’s lactation, induce depression, facilitate post-traumatic stress disorder [21]; and even can impact the type of delivery in the next pregnancy [22–24].

To enhance women’s awareness about normal childbirth, the Ministry of Health and Medical Education in Iran has reinforced helding childbirth preparation classes called “Physiological Childbirth Preparation” in selected hospitals and health centers since 2008. The Physiological Childbirth Preparedness classes have three parts: a) providing evidence-based information, b) counselling and c) facilitating practical skills. In the content of the first part concerning the related gestational age includes the anatomy of the female reproductive system, body adaptations during pregnancy, fetal growth and development, prenatal care, nutrition, personal hygiene and mental health, risk factors during pregnancy, benefits of vaginal delivery, pain relief methods, postpartum examinations and risk factors, and neonatal nursing. In the second part, counseling is provided in the form of questions and answers. In the third and final part, the following skills are taught practically: stretching exercises, posture correction, relaxation, massage, and breathing techniques for labor [25, 26].

The results of international studies demonstrated that participation in childbirth preparation classes can be associated with increased confidence in women for labor and childbirth [3], decreased labor pain and moderated the need for analgesics during labor due to reduced anxious feelings [17], promoted successful breastfeeding, and improved women’s relationship with the health care professionals [27, 28]. Some evidences have shown that participation in childbirth classes can increase women’s level of knowledge regarding childbirth [29–31].

In spite of many studies across the world, there is very limited number of studies in Iran that focus on evaluation of childbirth classes and its impact on women’s childbirth experience, maternal depression and anxiety, women’s satisfaction, and childbirth outcomes. There is no qualitative study in Iran to explore women’s perception on the impact of their childbirth preparation classes on their childbirth preparation. Employing both quantitative and qualitative approaches in a parallel convergent mixed study allows a better understanding of the phenomena around childbirth classes and its impact on women’s preparation and perception of childbirth experience [32]. Combining qualitative and quantitative data will provide better picture in understanding the situation and develop some recommendations to the policymakers and health planners to upgrade the quality of the childbirth preparation classes according to women’s need and desire.

### Study aim

The aim of the study is to explore and compare women’s childbirth experiences including childbirth fear, prenatal and postpartum depression, anxiety, and satisfaction with childbirth preparation classes among women with regular participation, irregular participation, and no participation in childbirth preparation classes. Women’s perceptions on the impact of participation in childbirth preparation classes on their childbirth experience will be explored through the qualitative arm of the study.

### The specific objectives of this study are to

1. Compare the mean scores for childbirth fear between the study groups (participation in 4 to 8 sessions, participation in 1 to 3 sessions, and no participation in any session of the childbirth preparation classes).
2. Compare the mean scores for prenatal anxiety between the study groups (participation in 4 to 8 sessions, participation in 1 to 3 sessions, and no participation in any session of the childbirth preparation classes).
3. Compare of the mean scores for prenatal and postpartum depression between the study groups (participation in 4 to 8 sessions, participation in 1 to 3 sessions, and no participation in any session of the childbirth preparation classes).
4. Compare of the mean scores for satisfaction with childbirth preparation classes between the study groups (participation in 4 to 8 sessions, participation in 1 to 3 sessions, and no participation in any session of the childbirth preparation classes).
5. Compare of the mean scores for childbirth experience between the study groups (participation in 4 to 8 sessions, participation in 1 to 3 sessions, and no participation in any session of the childbirth preparation classes).
6. Compare of the mean scores for knowledge of pregnancy and childbirth between the study groups (participation in 4 to 8 sessions, participation in 1 to 3 sessions, and no participation in any session of the childbirth preparation classes).
7. Determinate of psychometric properties of the Satisfaction with Childbirth Preparation Classes Questionnaire.
8. Exploration of women’s perceptions of the impact that participation in childbirth preparation classes will have on their childbirth experience.

## Method/ design

### Study design

**Fig. 1:**
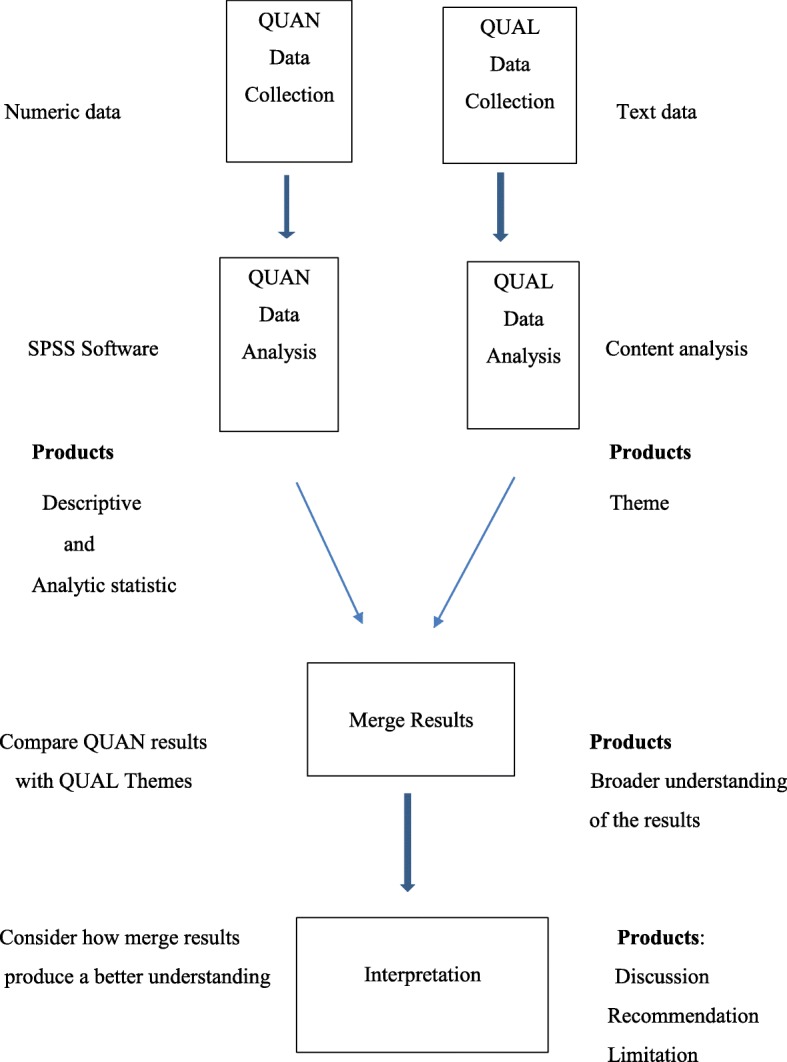
Study diagram

This mixed methods parallel-convergent study will be carried out in the quantitative (cohort study) and qualitative (content analysis) phases. In this study, the quantitative and quantitative data will be collected and analyzed simultaneously and independently [32]. The priority is the same and both data will have the same value in this study design. Data analysis will be performed separately and the results will be combined in the data interpretation stage (Fig. 1).

### Quantitative phase of the study

The quantitative phase of this study is a cohort type study. The target population in this phase will consist of primiparous pregnant women at the gestational age of 35–37 weeks divided into three groups (participation in 4 to 8 sessions, participation in 1 to 3 sessions, and no participation in any session of the childbirth preparation classes).

### Sample size and sampling method

The sample size in this study was calculated based on the scores for the variable of fear of childbirth using G-Power software. According to the results obtained by Najafi et al. on the relationship between participation in childbirth preparation classes and childbirth fear, and considering M_1_ = 37.29 (the mean score for fear in routine care group), SD_1_ = 9.55, M_2_ = 32.30 (the mean score for fear in the group participating in the classes), SD_2_ = 9.31, one sided α =0.05, and power = 90%, the calculated number of participants for each group will be 62. Assuming an attrition rate of 10%, the final sample size will be 68 in each group and 204 in all.

Tabriz is the second largest city in Iran and it has 20 health complexes. After obtaining ethics approval from the Ethics committee of Tabriz University of Medical sciences, six of health complexes will be selected randomly.

#### Recruitment

Potential participants will be invited in all health centers in the selected complexes. The research objectives and procedure will be fully explained to the selected women and, if they decide to take part in the study, written informed consent will be obtained from them. After consenting, they will be asked to fill out the demographic survey and antenatal questionnaire Women’s telephone numbers will be recorded and they will be informed that the researcher will call them 1 month after childbirth to ask them to visit the local health center in order to complete the postnatal survey and participate in an interview.

Based on the number of the primiparous pregnant women at the gestational age of 35–37 weeks at each center, a number of eligible women will be selected and divided into three groups (participation in 4 to 8 sessions, participation in 1 to 3 sessions, and no participation in any session of the childbirth preparation classes). The eligible participants will be matched in terms of education level, and sampling will continue to reach a sample size of 68 for each group and the total sample size of 204.

### Inclusion criteria

The inclusion criteria will be residence in Tabriz, primiparous women, and gestational age of 35–37 weeks.

### Exclusion criteria

The exclusion criteria will be multiple pregnancy, obstetric problems such as placenta previa, inclination to deliver babies via elective cesarean section, history of major diseases such as cardiovascular diseases, diabetes, chronic hypertension, preeclampsia, history of depression requiring medicine, and occurrence of important stressful event in the past 6 months such as death of a close relative, separation from the partner, etc.

### Scales and data collection

Antenatal data will be collected at recruitment by a demographic survey questionnaire, the W-DEQ (Version A) of Childbirth Fear Questionnaire, the Van den Bergh Anxiety Questionnaire, and the Satisfaction with Childbirth Preparation Classes Questionnaire, Knowledge regarding pregnancy and childbirth Questionnaire and the Edinburgh Depression Questionnaire.

Postnatal data will be collected by Obstetric and Childbirth Characteristics Questionnaire, the EPDS, and the Childbirth Experience Questionnaire at 4 weeks after childbirth. Each questionnaire and scale has been explained in more details.

Quantitative data will be collected using the Eligibility Checklist (Inclusion/ Exclusion Criteria), the demographic survey questionnaire, the Obstetric and Childbirth Characteristics Questionnaire, the Edinburgh Postpartum Depression Questionnaire, the W-DEQ Version A Childbirth Fear Questionnaire, the Mothers’ Knowledge of Pregnancy and Childbirth Questionnaire, the Childbirth Experience Questionnaire version 2.0 (CEQ2.0), and the Satisfaction with Childbirth Preparation Classes Questionnaire.

The demographic survey questionnaire including items such as age, spouse’s age, education level, and socioeconomic status will be completed at baseline. The Obstetric and Childbirth Characteristics Questionnaire including items such as delivery type and place will be completed 1 month after childbirth.

The Satisfaction with Childbirth Preparation Classes Questionnaire was developed by Lee et al. in 2006 to study the effects of Childbirth Preparation Classes (32). However, it has not yet been used in Iran. It will be used for the first time in this study, and its psychometric properties will be determined. The questionnaire consists of two parts: the first part includes 25 items in three separate sections on structure, process, and outcome of the classes. Participants should state their satisfaction using the 5-point Likert scale from 1 (not satisfied at all) to 5 (completely satisfied).

The Mothers’ Knowledge of Pregnancy and Childbirth Questionnaire was developed by the researcher based on the book “Pregnancy and Childbirth Training,” which is taught in childbirth preparation classes. It includes 20 four-answer choice questions with one true option. Each correct answer is given one point.

To assess childbirth fear, the W-DEQ (Version A) Childbirth Fear Questionnaire will be used. This questionnaire, designed by Wijma et al. in 1998, has 33 questions and measures prenatal fears and expectations. The pregnant mothers will specify their personal feelings and cognition based on the 6-point Likert scale (from zero for never to 5 most of the time). The total score obtained from the sum of the scores for all items will range from zero to 165. The total score of 100 is the cutoff point and higher scores will show greater childbirth fear [33].

The Pregnancy Related Anxiety Questionnaire (PRAQ), developed by Van den Bergh (1999), consists of 34 items and covers 5 areas. Huizink et al. identified the areas with high factor loadings by conducting a factor analysis, classified the 5 areas into the 3 areas of childbirth fear, fear of delivering infants with mental and physical disabilities, and concern about their appearance and reduced the number of items from 34 to 10. This questionnaire is scored using a Likert scale from 1 (totally disagree) to 5 (totally agree). The higher the score is the greater the anxiety of the subject will be [13].

The Edinburgh Pregnancy Depression Questionnaire, this questionnaire, developed by Cox et al. in 1987, is used to measure prenatal and postpartum depression. It consists of 10 four-answer choice questions. In some items, the options are ordered from low to high intensity (items 1, 2, and 4) and vice versa in others (items 3, 5–10). The items receive scores from zero to three according to the intensity of symptoms and the scores for all the items are summed up to yield the total score of the individual that can vary from zero to 30. Mothers with scores above the threshold of 12 have varying degrees of depression [34]. The psychometric properties of this questionnaire were determined by Montazeri et al. [35].

The Childbirth Experience Questionnaire with 22 items measures the childbirth experience of primiparous women. It covers 4 areas: personal capacity (sense of control, personal feeling about childbirth and labor pain), professional support (provision of information and midwifery care), perceived safety (feeling of safety and childbirth memories), and participation (the woman’s ability to change her position and movements and to relieve pain during labor and childbirth). The questionnaire includes 19 four-answer choice questions and 3 questions are scored using the Visual Analogue Scale (VAS). The answers are: totally agree (score of 1), often agree (score of 2), often disagree (score of 3), and totally disagree (score of 4). Scores given to items using the VAS are converted to scores from 1 to 4: scores of 0–40 (score of 1), 41–60 (score of 2), 61–80 (score of 3), and 80–100 (Score of 4). Items with negative concepts (experiencing severe pain, feeling tired, feeling fear, and having bad memories) are given negative scores. High average scores in this tool suggest a more positive childbirth experience [36].

### Data analysis

The collected quantitative data will be analyzed in SPSS 24. The socio-demographic and obstetric characteristics will be described employing descriptive statistics including frequency (percent) and average (standard deviation) for normal data, and median (percentile 25 to 75) for abnormal data. The variables of knowledge, fear of childbirth, depression, anxiety, satisfaction with childbirth preparation classes, and childbirth experience will be compared between the study groups using one-way ANOVA in bivariate analysis and employing multivariate linear regression with control of socio-demographic and obstetric and childbirth characteristics in multivariate analysis.

### Qualitative study

The qualitative phase of this study will be performed simultaneously with and independently from the quantitative phase to explore the women’s perceptions of the impact of participation in childbirth preparation classes on their childbirth experience. The two phases receive the same priority and their data will have the same value. Data analysis is usually performed separately and the results are combined in the data interpretation stage.

### Sampling method and data collection

In this study, the participants in the qualitative phase will be women who have participated in childbirth educational classes. They will be studied individually through in-depth, semi-structured interviews with open questions 1 to 2 months after childbirth to collect the qualitative data, the methods of obtaining valid data and of focusing on research questions will be reviewed with members of the research team. The interviews will start using the pre-defined questions and continue with more in-depth items, such as “what do you mean? Why? Can you explain further? Can you give an example?” to explore the depth of their experience. During the interview, the researcher will record nonverbal data of the participants, such as tone, facial expression, and position, in a specific sheet, along with the time and place of the interview. The sampling will continue until data are saturated.

### Data analysis

Qualitative data will be analyses based on content analysis using a conventional approach. In this approach, data analysis begins by repeatedly reading the transcribed texts to have a complete understanding of them. The texts are then read word by word to extract codes. Initially, objective words of the text that seem to embrace the main concepts are determined. The researcher continued digging the text by taking notes from the initial analysis until the major codes will be extracted. In this process, the code labels reflecting more than one key thought will be directly extracted and specified. Then, the codes will be categorized based on their difference and/or relationships. Ideally, 10–15 categories will be considered sufficient for categorization of a huge amount of data. The main advantage of qualitative content analysis based on a conventional approach is that direct information is obtained from the study without imposing predetermined categories or theories [37].

## Discussion

Fear of childbirth and of not being to tolerate labor pains are the most important reasons why women prefer caesarian- section without any medical reason [38–40]. Due to the increase in the rate of cesarean- sections around the world, international policies and approaches are used to encourage women to choose vaginal birth [41]. Primiparous women feel greater stress in adapting to their new role as a mother who takes care of her infant. These mothers are more inclined to participate in childbirth preparation classes [42]. Lack of knowledge and unpreparedness of women can lead to emergence of anxiety and complications followed by ever-increasing medical interventions [43]. Evidence has also shown that participation in childbirth classes reduces anxiety about delivery and generates suitable response to pain [1].

Although, department of health in Iran promotes and supports childbirth preparation classes, there is no rigorous study that has comprehensively explored the details of these classes and the impact on women’s preparation and childbirth experiences. This will be the first study in Iran that assesses the childbirth preparation classes comprehensively. It is expected that the results of the study will be effective in improving the health of pregnant mothers and their newborns.

## Data Availability

Not applicable.

## Abbreviations

CEQ: Childbirth experience questionnaire
EPDS: the Edinburgh Postpartum Depression Scale
PRAQ: Pregnancy Related Anxiety Questionnaire
W-DEQ: Wijma Delivery Expectancy/Experience Questionnaire
